# Norepinephrine infusion in septic shock

**DOI:** 10.4103/0972-5229.40952

**Published:** 2008

**Authors:** P. S. R. K. Haranath

**Affiliations:** Director of Medical Education A. P. (Retired) and Former Professor of Pharmacology

Sir,

The paper ‘Comparison of norepinephrine and dopamine in the management of septic shock using impedance cardiography’[1] in the December 2007 issue is interesting. The authors conclude that norepinephrine is better than dopamine in septic shock. In this connection, I wish to draw attention to our experiments in anesthetized dogs where infusion of norepinehrine 10 μg/min/kg given i.v. produced immediate rise of blood pressure. But the rise was not sustained, despite continued infusion and was followed by hypotension and finally death.[2] The tachyphylaxis could be due to altered receptor sensitivity, cardio vascular reflex changes, or release of vasodilator substances like prostaglandins from underperfused organs. Preliminary injection of indomethacin (a prostaglandin synthesis inhibitor) 10 mg/kg bolus 15 min before the norepinephrine infusion increased duration of pressor effect. This information may be clinically useful.
